# Supplementary material for: Beyond convexity—Contraction and global convergence of gradient descent
Source: PLoS One. 2020 Aug 4;15(8):e0236661. doi: 10.1371/journal.pone.0236661 (PMC7402485; doi:10.1371/journal.pone.0236661)
Supplement: S1 File — (PDF) [file pone.0236661.s001.pdf]

Supporting Information

Proof of Theorem 1

Proof. Recall that α-strong geodesic convexity of $f(\mathbf{x}, t)$ in the metric $\mathbf{M}(\mathbf{x})$ (for each t) is equivalent to the Riemannian Hessian of f, denoted $\mathbf{H}(\mathbf{x}, t)$, satisfying:

$$\mathbf{H}(\mathbf{x}, t) \succeq \alpha \mathbf{M}(\mathbf{x}) \quad \forall \mathbf{x}$$

We now show that this property is exactly the same as contraction of the natural gradient dynamics (9) in the metric $\mathbf{M}(\mathbf{x})$. Specifically, given $\mathbf{h}(\mathbf{x}, t)$ from (9), and defining

$$\mathbf{Q} = \mathbf{M}\left(\frac{\partial \mathbf{h}}{\partial \mathbf{x}}\right) + \left(\frac{\partial \mathbf{h}}{\partial \mathbf{x}}\right)^{\top} \mathbf{M} + \dot{\mathbf{M}}$$

we show that $\mathbf{Q} = -2\mathbf{H}$, thus proving the theorem.

In coordinates, entries of $\mathbf{H}$ are given by

$$H_{ij} = \partial_{ij} f - \Gamma^k_{ij} \left(\partial_k f\right)$$

where Γ^k_{ij} denotes the Christoffel symbol of the second kind

$$\Gamma^m_{ij} = \frac{1}{2} M^{mk} \left(\partial_j M_{ik} + \partial_i M_{jk} - \partial_k M_{ij}\right)$$

and the usual Einstein summation convention is applied (implying, e.g., a sum over k in the above).

Consider the partials of the natural gradient system,

$$\begin{aligned} \partial_j h_k &= \partial_j \left[-M^{k\ell} \left(\partial_\ell f\right)\right] \\ &= -M^{k\ell}(\partial_{\ell j} f) + M^{kr}(\partial_j M_{rs}) M^{s\ell}(\partial_\ell f) \end{aligned}$$

Using this result

$$\begin{aligned} Q_{ij} =& M_{ik}(\partial_j h_k) + M_{jk}(\partial_i h_k) - (\partial_k M_{ij}) M^{k\ell}(\partial_\ell f) \\ =& - M_{ik} M^{k\ell}(\partial_{j\ell} f) + M_{ik} M^{kr}(\partial_j M_{rs}) M^{s\ell}(\partial_\ell f) \\ & - M_{jk} M^{k\ell}(\partial_{i\ell} f) + M_{jk} M^{kr}(\partial_i M_{rs}) M^{s\ell}(\partial_\ell f) \\ & - (\partial_k M_{ij}) M^{k\ell}(\partial_\ell f) \end{aligned}$$

Noting that $M_{ik} M^{kj} = \delta_{ij}$, with δ_{ij} the Kronecker delta,

$$\begin{aligned} Q_{ij} &= -2\partial_{ij} f + M^{s\ell} \left(\partial_j M_{is} + \partial_i M_{js} - \partial_s M_{ij}\right) \partial_\ell f \\ &= -2 \left(\partial_{ij} f - \Gamma^k_{ij}\, \partial_k f\right) \\ &= -2 H_{ij} \end{aligned}$$
